# Patient experience of the acute post‐surgical period following total laryngectomy during the COVID‐19 era

**DOI:** 10.1111/1460-6984.12709

**Published:** 2022-04-11

**Authors:** Laura‐Jayne Watson, David Hamilton, Joanne M. Patterson

**Affiliations:** ^1^ Speech & Language Therapy South Tyneside & Sunderland NHS Foundation Trust Sunderland Royal Hospital Sunderland UK; ^2^ Otolaryngology Head and Neck Surgery Freeman Hospital, Newcastle upon Tyne Hospitals NHS Foundation Trust Newcastle upon Tyne UK; ^3^ School of Health Sciences, Institute of Population Health/Liverpool Head and Neck Centre University of Liverpool Liverpool UK

**Keywords:** enhanced recovery after surgery (ERAS), laryngectomy, patient experience

## Abstract

**Background:**

Total laryngectomy (TL) results in permanent functional changes requiring rapid development of complex new skills. A significant portion of this learning happens in the acute post‐surgical stage. There is increasing interest in enhanced recovery after surgery (ERAS) protocols in TL; however, implementation has been difficult. COVID‐19 has placed significant pressures on acute services, requiring rapid service changes for TL patients.

**Aims:**

To understand the acute patient experience of having a TL both before and during COVID‐19.

**Methods & Procedures:**

Semi‐structured interviews using a pre‐designed topic guide were conducted with 10 people who had undergone a TL within the last 2 years. Participants were recruited by their speech and language therapists using purposive sampling. Braun and Clarke's iterative approach to data collection and thematic analysis was used to generate key themes from the data.

**Outcomes & Results:**

Thematic analysis identified four main themes: (1) pre‐operative information‐giving: ‘it was just words’; (2) decision‐making influences: ‘I just wanted them to get it all out and get it over with’; (3) coping with adjustment to the new normal: ‘this is part of me now’; and (4) the importance of relationship‐building: ‘when you've had something like this, you need some care and understanding’.

**Conclusions & Implications:**

The need for an individualized approach to TL intervention which incorporates medical and psycho‐social approaches from pre‐treatment to acute discharge is vital. ERAS models should be reviewed to shift beyond the medical model alone. Rapid service changes due to COVID‐19 did not contribute any major changes to the acute patient‐reported experience.

**What this paper adds:**

**What is already known on the subject**

- We know that ERAS protocols have the potential to improve patient outcomes following TL. However, the research does not consider anything other than the early oral feeding debate and it has therefore been difficult to implement ERAS protocols in current service models. COVID19 required head and neck cancer services to make quick changes to surgical pathways, with the potential that some ERAS protocols had been adopted inadvertently. In order to understand the impact of this, we need to understand the patient experience following TL both before and during COVID19.

**What this paper adds to existing knowledge**

- This paper used qualitative interviews to understand the acute patient experience following TL both during and before COVID19. Findings from these interviews highlighted that people were on the most part, well prepared for the functional changes they would experience after surgery. However, people felt there were gaps in service delivery at the pre‐treatment and early discharge home period. Overall, the gaps identified were from a more psycho‐social need suggesting that future ERAS models of care should consider both medical and psycho‐social principles to enhance patient experience and outcome.

**What are the potential or actual clinical implications of this work?**

- Pre‐treatment services provided to people who have a TL could be reviewed to help maximize long‐term adjustment to life. Areas which could be reviewed include the method and mode of information delivery. Further work needs to be done in partnership with community services to improve the immediate discharge home experience.

## BACKGROUND

Total laryngectomy (TL) is a treatment option for some people with advanced laryngeal cancer (Bozec et al., 2020), but it can have devastating consequences to everyday functions including loss of a naturally produced voice, and changes to eating, drinking, swallowing and breathing. People after a TL therefore need to learn a set of complex new skills to adapt to their new communication method, eating and breathing function. A significant portion of this learning takes place in hospital after the surgery, which can often be distressing and traumatic (Perry et al., 2015).

Recently, there has been increasing interest in enhanced recovery after surgery (ERAS) pathways in head and neck cancer (HNC) to facilitate early and safe discharge home. ERAS originated from general and colorectal surgery with pathways in these specialities demonstrating improved long‐term patient outcomes (Muller et al., 2009). ERAS pathways in TL have primarily focused on early oral feeding (Boyce & Meyers 1989; Seven et al., 2003; Prasad et al., 2006; Bannister & Ah‐See 2015; Aires et al., 2015) to reduce overall hospital length of stay without adversely impacting on patient outcomes, for example, increased risk of post‐operative infection. The positive outcome from these studies has concluded that there is no increased risk of pharyngo‐cutaneous fistula formation (Seven et al., 2003; Prasad et al., 2006; Bannister & Ah‐See 2015; Aires et al., 2015). However, the quality of the studies conducted is variable, for example, a lack of participant homogeneity and limited use of quality appraisal methods (Watson & Ewers 2020). It is therefore unclear whether the introduction of early oral feeding after TL has the expected impact on hospital length of stay. However, there is potential that these protocols could reduce hospital length of stay, as well as result in cost savings and positively influence patient recovery.

Consequently there is controversy around the implementation of early oral feeding regimes (Watson & Ewers 2020), leaving clinicians with unclear guidance for implementation into clinical pathways. Additionally, other elements of care such as early stoma education are not adequately considered in current ERAS laryngectomy literature (Seven et al., 2003; Prasad et al., 2006; Bannister & Ah‐See 2015; Aires et al., 2015). As a result, there are significant challenges in successfully implementing ERAS protocols in the acute post laryngectomy period; and it may be that ERAS in laryngectomy care needs to shift focus beyond the medical model alone. A potential option to consider is a ‘multiphasic prehabilitation’ model (Santa Mina et al., 2021). Whilst novel in its approach; multiphasic prehabilitation advocates a move beyond the medical model alone and incorporates principles of survivorship into prehabilitation models of care, that is, maximizing physical, social, psychological and vocational functioning within that individual's capacity following cancer treatment (Cromes & Fred 1978). In practice, this would require a multidisciplinary team (MDT) of professionals working collaboratively with individuals to assess and implement physical, social, psychological and vocational interventions at repeated time points from diagnosis to optimize health, maximize outcomes and achieve rapid and safe recovery after surgery.

Whilst ‘multiphasic prehabilitation’ could be a potential option to consider for TL patients, quick changes to acute HNC surgical pathways, which includes patients undergoing TL, have been made due to the COVID‐19 pandemic, for example, commencing oral intake earlier, starting stoma care competencies quicker. Particularly, there has been significant emphasis on reducing hospital length of stay to facilitate early, safe discharge home to reduce the coronavirus transmission risks to high‐risk HNC patients (Day et al., 2020). Patients undergoing a TL are particularly vulnerable in the acute phase of treatment due to increased risk of infection and there are significant threats to HNC services as a result of COVID‐19 (Patterson et al., 2020). Head and neck teams were recommended to avoid primary tracheoesophageal puncture (TEP) to reduce the risk of post‐operative problems (BAHNO, 2020) including wound complications (Panwar et al., 2017) which would increase hospital length of stay. Consequently, local service delivery models have rapidly changed to incorporate these recommendations, with the majority of patients not being offered primary TEP, the ‘gold standard’ for communication rehabilitation (Blom et al., 1998; Hutcheson et al., 2012; Sethi et al., 2014). People having a TL during COVID‐19 are left without a voice and therefore dependent on alternative communication methods which can sound unnatural and are often difficult to learn effectively, for example, electro‐larynx, oesophageal speech.

It is therefore likely that there has been a possible unanticipated shift towards some principles of ERAS in laryngectomy care as a result of COVID‐19 recommendations. For example, patients may learn stoma care competencies earlier, recommence oral intake earlier or be discharged sooner. The result of this rapid change in service provision on patient outcome, hospital length of stay, patient‐reported quality of life, and experience is currently unknown. It is imperative that the rapid changes to acute laryngectomy service provision is evaluated from the patient's perspective in order to understand the impact of this change on both the patient experience and our clinical services offered to TL patients in the COVID‐19 era (Patterson et al., 2020).

## AIMS

To explore the acute patient experience following TL both before and during the COVID‐19 pandemic.

## METHODS AND PROCEDURES

### Study recruitment

Participants were recruited from a single unit in the North East of England and were eligible for recruitment to the study if they had had a TL within the last 2 years. Participants were recruited to the study via purposive sampling. Purposive sampling was used to ensure collection of data rich information (Patton 2002) from a group of participants who were knowledgeable about the study question (Cresswell & Plano Clark 2011). The first five participants had their TL during the COVID‐19 pandemic, had completed all of their cancer treatment that is, adjuvant radiotherapy and used any communication method for example, silent articulation, electro‐larynx. The next three participants had had their TL before the COVID‐19 pandemic, had completed all their cancer treatment and used any communication method. This allowed comparison of the patient experience before and during COVID‐19 which meets the aim of this study. The final two participants had just had their surgery that is, were within the first two weeks of discharge from hospital and therefore may not have completed all their cancer treatment. These participants were approached to capture the immediate reported inpatient experience to determine whether time had any impact on information recall. Interviews were stopped after this as no new codes or themes were generated.

Patients with active or recurrent disease, receiving palliative care or with cognitive issues making it difficult to recall their experiences, participate in an interview or which would impact on capacity were excluded. Demographic information including age, gender, cancer site/stage/treatment, time post‐surgery and social deprivation level using the English Indices of Social Deprivation 2019 (see www.gov.uk) was collected for all participants. The indices rank each small area in England from most—least deprived based on the following seven domains: income, employment, education, health, crime, barriers to housing and services, and living environment. Ranking is based on geographical location using a scale of 1 to 10: those in group one being in the 10% most deprived areas of the UK and those in group 10 being in the 10% least deprived areas.

All eligible participants were approached by a speech and language therapist between November 2020 and May 2021 and were given a patient information sheet describing the study and a consent form (see Appendix S1 in the additional supporting information). These were either given in person at an outpatient appointment or posted to participants with a pre‐addressed stamped envelope enclosed to return completed forms prior to the interview. The lead researcher (L.J.W.) contacted all potential participants to answer questions about the study and arrange a convenient time for the interview if they wished to take part. Participants were not obliged to take part in the study and could withdraw at any time. Some participants had received clinical intervention from the researcher so for the purpose of the interview, specific information about the researchers’ role and interest in the research area was provided to improve transparency and frankness in participants’ responses (Tong et al. 2007).

### Data collection

All participants were offered either face‐to‐face interviews alongside a cancer follow‐up appointment or virtual interviews via telephone or NHS‐attend, a secure video‐conferencing platform, in a private room at the unit. Participants were invited to bring a partner/friend to the interview if they wished but results from the partner/friends’ responses were not included in the final analysis. Written consent was obtained prior to the interview.

Interviews were semi‐structured as they allow the researcher to understand an individual's perspective by exploring their thoughts, feelings and beliefs while probing very personal and sensitive experiences (DeJonckheere & Vaughn 2019). A topic guide (see the Appendix in the additional supporting information) was designed by two of the research team, using the literature base and clinical expertise as the basis, and reviewed by a patient representative. This topic guide was revised after interim analyses with the research team. This follows an iterative process (Srivastava & Hopwood 2009) to ensure depth and richness of data collection and analysis based on participants’ responses to ensure the aims of the research were met.

Interviews were audio‐recorded using Audacity software and written field notes were taken by the interviewer to support the recording for example, if participant used silent articulation as their main communication method. Interview length ranged from 45 to 80 min and on average lasted 60 min. All interviews were transcribed verbatim, allocated a number to maintain confidentiality and stored securely with the audio‐recording on an NHS computer in a password protected file.

This study was registered and approved as a service evaluation by the research and development department at the unit in August 2020.

### Data analysis

Reflexive thematic analysis as described by Braun & Clarke (2013) was used for interview analysis. This approach to qualitative data analysis is described as being both accessible and flexible by understanding, exploring and interpreting individuals’ experiences through organization of data into a pattern of shared meaning. Braun and Clarke's (2013) approach does not fit data into a pre‐existing framework (Fereday & Muir‐Cochrane 2006) and themes do not ‘organically emerge’, nor are identified prior to data collection. Instead, themes are interpreted, generated and discovered to reflect the active role of the researcher in the analysis (Braun & Clarke 2020). This acknowledges the importance of the researchers’ subjectivity within data analysis (Braun & Clarke 2019) which in this instance is the researchers’ clinical expertise in TL. In Braun and Clarke's approach, there are six different orientations which can be used in thematic analysis: inductive, deductive, semantic, latent, critical and constructionist. For this research, an inductive, semantic and critical approach was taken. As described by Braun and Clarke, this approach to thematic analysis is directed by the content of the data with theme development reflecting the explicit content of the data and reporting focusing on a reality evident in the data.

Following decision regarding data orientation, the recursive six step framework as described by Braun and Clarke was used as follows: data familiarization, generating initial codes, searching for themes, reviewing themes, defining themes, writing up. All interview transcripts were read multiple times by the lead researcher (L.J.W.) to generate initial conceptual codes. A list of codes was developed which were then grouped together to search for initial themes. This was done on an interim basis after every two to three interviews. Initial themes were discussed virtually on multiple occasions with the research team; and re‐reviewed alongside the transcripts to explore whether any detail was missing and ensure rigor, reliability and validity in data analysis. Final themes were established following this process and defined using a pattern of shared meaning. Themes were then defined and named to shape the focus of each theme and ‘tell the story’ (Braun & Clarke 2013). A copy of the interview findings was sent to all participants for feedback. Seven participants returned this feedback and agreed with the interpretation of the interview data.

## OUTCOMES AND RESULTS

A total of 17 patients underwent TL at the unit within the 2‐year inclusion period. Six participants were ineligible due to either palliative disease status or being under active investigation for other health conditions. All eligible participants were approached and 10 consented to participate; five participants had a partner present for the interview. One participant did not respond to the study invitation.

Table 1 summarizes participants’ characteristics. The sample consisted of seven males and three females, with a median age of 63 years. Participants used a range of communication methods including writing, silent articulation, electro‐larynx, oesophageal speech and tracheoesophageal voice. Five participants had a glottis cancer site. Five participants had a cancer stage of TI–II and five had a staging of TIII–IV. Four out of five participants in the TI–II cancer staging group had residual or recurrent disease. Over half the participants received combined modality treatment: six having surgery followed by chemo/radiotherapy and three needing salvage surgery for recurrent/residual disease. All participants who had salvage surgery had a flap reconstruction as part of their surgery. Six participants were within their first year post‐TL. Seven participants lived within the top four most deprived areas in the UK.

**TABLE 1:** Participant characteristics

Variable	*N*	%
*Sex*		
Male	VII	70
Female	III	30
*Age at interview (years)*		
< 50	II	20
50–59	I	10
60–69	III	30
70+	IIII	40
*Cancer site*		
Larynx	V	50
Supraglottis	III	30
Pyriform fossae	II	20
*Cancer stage*		
I	I	10
II	IIII	40
III	I	10
IV	IIII	40
*Months since surgery*		
< 1	II	20
2–3	I	10
4–6	I	10
7–12	II	20
13+	IIII	40
*Treatment received*		
Primary surgery alone	I	10
Primary surgery + adjuvant chemo/radiotherapy	VI	60
Salvage surgery	III	30
Free flap		
Yes	IIII	40
No	VI	60
*Length of stay (days)*		
1–7	0	0
8–14	V	50
15+	V	50
*Level of social deprivation*		
1	II	20
2	IIII	40
3	0	0
4	I	10
5	II	20
6	0	0
7	0	0
8	I	10

Four equally important, interrelated themes were generated from the analysis. These describe significant factors and principles which influence the post‐operative inpatient acute experience following TL. Two themes highlighted patients early decision‐making processes about TL based on their own personal values and the information they were provided with; and the other two themes demonstrated how both internal and external factors shaped early post‐operative recovery and initial coping to life with a TL. Themes included: (1) pre‐operative information‐giving: ‘it was just words’; (2) decision‐making influences: ‘I just wanted them to get it all out and get it over with’; (3) coping with adjustment to the new normal: ‘this is part of me now’; and (4) the importance of relationship‐building: ‘when you've had something like this, you need some care and understanding’. There was little difference between participant responses when comparing those who had their surgery before COVID‐19 with those who had their surgery during COVID‐19; therefore limited weight was given to this area in the final thematic framework.

**FIGURE 1:**
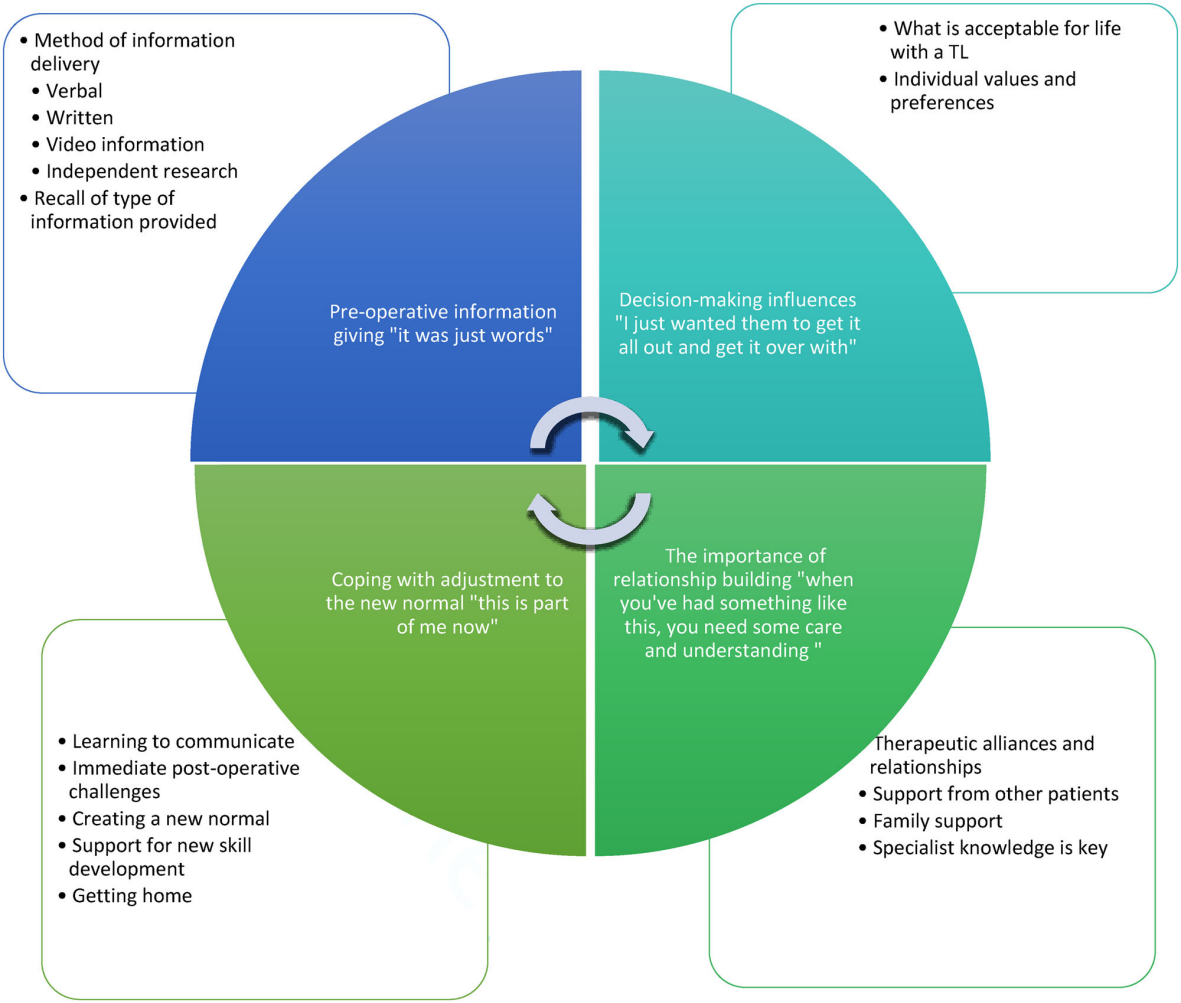
Thematic framework

Figure 1 demonstrates the thematic framework developed from analysis, representing the four key themes and their subthemes.

### Theme 1: Pre‐operative information‐giving: ‘it was just words’

All participants discussed the information they were provided with before their surgery, which they mainly recall being delivered verbally by their consultant surgeon: ‘I was told about the operation and that was all’ (P8, M).

Only a few participants could remember meeting other members of the MDT, for example, clinical nurse specialist, speech and language therapist, at this stage. Participants recalled a lot of verbal information provided about surgical details, immediate post‐operative changes to communication, breathing and swallowing, and options for communication after their surgery. However, some participants were overloaded with information before their operation:

Mrs. X says that they didn't know whether I'd have a voice box put straight in erm … it all depended on when they got further down and you know they could see what was what and whether they needed to take any tissue from either the top of me leg or me arm. (P4, F)

Other forms of information were provided for example, written and video: ‘I got a CD … well I knew what I was going in for put it that way’ (P1, M). However, participants did not talk about anything other than the key surgical information being provided in these forms. When written information was provided, most participants said they either did not read it or found the language too complex to process the information: ‘In some parts they explained it okay but in other parts there were some words … bigger words I couldn't understand so I had to ask about them’ (P7, F).

Two participants looked for information themselves online: ‘I looked on YouTube … I wanted to know what to expect’ (P10, F).

No participants discussed information about what life might be like for them in the longer term after TL when revisiting the information provided to them pre‐operatively.

### Theme 2: Decision‐making influences: ‘I just wanted them to get it all out and get it over with’

All participants discussed the influences which played a role in enabling them to make their decision about proceeding with a TL. This consisted of participants weighing up what was important for their life after surgery; with all participants talking about their personal preferences in their life, for example, the importance of family. All participants talked about the need to survive and as a consequence, the need for survival was often the main priority for participants when making decisions about their treatment: ‘I wanted to be here for V (husband) … it wasn't my time to go yet’ (P7, F).

For some participants, this decision‐making process was based around both survival and getting back to ‘normal’ life as quickly as possible after the operation:

Beforehand I was worried about the cancer, I just wanted it cut … I thought I was going to go back to normal … I just wanted to be back home, to get back with my son and family. I just wanted to get it out of the way. (P2, M)

Participants also talked about what was going to be an acceptable trade‐off for their life after surgery, for example, a willingness to accept losing their voice permanently in order to survive: ‘I was saying to Y (wife), I've still got me good health, I can see, I'm fit, I can walk, it's only going to be the voice’ (P5, M). However, the majority of participants had not considered the reality about how life would be from a practical, physical and emotional perspective in the longer term following their discharge from hospital. Participants mainly talked about how they got to grips with key skills after their operation so that they could get home quickly; they did not think about their life beyond this period of time.

Interviewer: Did you want to know what life would be like after?

Participant: It didn't really cross me mind … I just wanted to get out of hospital as quickly as possible. (P9, M)

This theme is strongly linked with ‘Theme 1’ as participants seemed to use the information provided from their consultant surgeon to help inform their decision about survival:

I think I did ask which was erm best for the success thing … wey how many average out of a hundred sorta thing survive and sorta thing … he didn't say how may out of a hundred, he just said there's more success with the one I've got so that's what I opted for. (P5, M)

### Theme 3: Coping with adjustment to the new normal: ‘this is part of me now’

When talking about their time in hospital after the operation, participants described how they started to adjust to their ‘new normal’: ‘I was a bit shocked at first, then I learnt to live with it’ (P9, M). Whilst within this ‘new normal’ participants talked about the anticipated changes in communication, swallowing and breathing that they were prepared for; they also talked about their practical adjustment, such as how they began to prepare for the transition from hospital to home: ‘You got into a routine and you realise that routine, if it wasn't here, it would be very difficult at home’ (P3, M).

Participants described how they learnt to communicate, look after their stoma, and swallow again; with an overall feeling that they knew they would have to do this. Adjusting to communication was described as being most difficult by the majority of participants, followed by stoma management with swallowing being of lesser need.

There was a wide range in experiences, particularly in how participants learnt to communicate again, with some participants finding the experience challenging: ‘I was using a board to write things on and that, that was very stressful … it was more stressful than I thought it was going to be’ (P1, M). Others found this experience more rewarding: ‘I was over the moon with that … very excited about it … actually being able to speak to somebody rather than writing notes down’ (P3, M talking about electrolarynx use).

The way in which participants coped with these changes was individualized, for example, some participants took photographs of their stoma as a way to initially look at themselves post‐operatively. How participants coped was affected greatly by their mood, as some participants reported that they did not have the motivation to engage in learning these new skills in the early days after their operation: ‘first week I just couldn't be chewed … I mean I did get up and get washed but it was like “agh”’ (P2, M).

All participants said that they needed some support to develop these skills, with the level of support varying dependent on their level of adjustment to the ‘new normal’: ‘the nurses showed iz … they used to take it out on a morning and a night and then I would do it four or five times a day’ (P8, M explaining laryngectomy tube removal/stoma care).

Participants said they also had a strong focus on trying to cope with the adjustment to their ‘new normal’ so that they could get home, usually to their families, and often had a specific and individual goal for their length of hospital stay. Some participants were able to have a staged return to home, for example, day leave from hospital: ‘they started letting iz go out for a couple of hours a day, maybe once or twice a week and then stayed over home for a night and that was absolutely great ’coz I missed me family’ (P4, F).

These participants reported that this helped them consider what life would be like beyond hospital, for example doing their stoma care at home. However, the majority of participants said that they had given little thought to their return to home, that is, how this ‘new normal’ would be integrated into what their everyday life looked like pre‐operatively. Some participants who had surgery in the COVID‐19 era commented on the lack of face‐to‐face visiting during their post‐operative inpatient stay, but this was more from a lack of interaction perspective rather than an adjustment issue:

I like meeting people, I like talking to people and I like socialising. So to lie there and sit there with nobody there it's bound to affect you in some way and it affected me so visitors would've been fantastic. (P3, M)

### Theme 4: The importance of relationship‐building ‘when you've had something like this, you need some care and understanding’

The importance of relationships was discussed extensively by all participants, specifically those relationships with healthcare professionals, peers and family: ‘You need family to talk too … it's a big operation’ (P10, F).

Participants described the positive therapeutic relationships formed with healthcare professionals and how these relationships made them feel as a person. This relationship and interactions between healthcare professionals and participants helped participants to feel normal, not just another patient who was going to have or had had a TL:

there was some nice characters of the nurses … funny … make a joke with iz … just didn't look at iz any different to any other patient which was really good … It made iz feel normal … it made iz forget. (P2, M)

Pertinent to this relationship was the level of expertise that healthcare professionals displayed, with some participants feeling negative about the lack of specialist support once they were at home:

I have rang her once when I needed some things for the nebuliser … that was a waste of time because they haven't even got them, they sent iz an spacer wey I says ‘that's no good, I've got a stoma I cannit even put it over me mouth. (P4, F talking about community support)

Some participants described the relationships that they formed with other patients on the ward, and the positive impact this had on their recovery by facilitating the adjustment to their new normal:

You know X she would say ‘are you alright Y?’ and you know I would try and I would say ‘I'm, I'm a‐a‐a‐alright X’ you know that's the way I was sounding. She says ‘Y it's great to hear you flower’ and erm I really took to her well you know X. (P4, F)

A sense of togetherness and connection came through strongly for those participants who found these relationships helpful; and having a natural environment for this to happen in seemed to help for example, swapping newspapers in the day room. All participants were beyond grateful for the relationships they had with their families which supported them through all time points in their journey pre‐ and post‐TL: ‘Me Mam and Dad were watching the bairns … I knew I had good family support’ (P6, M).

Family relationships were described as being important for all participants, whether that be a spouse/partner, parent or child.

## CONCLUSIONS AND IMPLICATIONS

This study provided a unique insight into the early post‐operative phase following TL from the patient's perspective. Four equally weighted themes were generated from qualitative data analysis, with the key points from these being: information provision, decision‐making influences, coping with adjustment to the new normal and relationships with family, friends, healthcare professionals and other patients. The key message between these themes from participants was the need for a person‐centred approach to both the practical and psycho‐social needs as a TL, particularly at the pre‐operative and early discharge home phase. Qualitative information about information provision at the pre‐operative stage was an incidental finding from this study. However, this does have direct implications on how people are prepared for what will happen during the acute inpatient period after TL; and from the experiences described, participants were on the most part, adequately prepared for their post‐operative time in hospital.

As well as being adequately prepared for their post‐operative time in hospital; the majority of participants were prepared for the anticipated changes to communication, swallowing and breathing. Participants were given practical and functional verbal information during their pre‐operative preparation which fits with standard practice in UK clinical services (NICE Improving Outcomes Guidance, 2004). However, for some participant's complex information provided appeared to overload them, which questions how useful this information has been in supporting them beyond the surgical details, acute rehabilitation and potentially their understanding of life‐long changes following a TL. This finding has been explored in other qualitative literature (Llewellyn et al., 2006; Brockbank et al., 2015) which advocates for personalized pre‐treatment information delivered at an individual's own pace and which incorporates information about longer term outcomes and support; for example, how the long‐term effects of treatment will impact an individual's work ability and quality of life.

This level of information overload reported by participants is likely due to the multitude of complex information provided verbally at a highly stressful time. Although written information was provided, the majority of participants either did not read this or found the information too complex to process. A potential reason for this could be linked to low health literacy levels which are reported to be prevalent in 14% of HNC survivors (Nilsen et al., 2020) and also in areas with high levels of social deprivation (Simpson et al., 2020). Seven of the 10 participants in this study lived in the top four most socially deprived areas in the UK, hence it is possible that issues with health literacy have contributed to information overload for these participants. It is also possible that a reduced capacity to process information at a highly stressful time has contributed to this information overload; a concept which has been highlighted in qualitative work by Govender et al. (2019) whilst investigating the use of video‐animation in patients with dysphagia following HNC. Participants in Govender et al. also experienced information burden and cognitive overload, with findings recommending the use of multi‐modal, personalized information to meet the individual needs of each patient. Although this has not been tested, this concept could be considered in information provision, particularly at the pre‐operative stage, for people having a TL.

Strongly linked to information overload was the participants’ ability to navigate through the information in order to make decisions about their treatment and care. Often treatment decision was centred on survival, with participants not fully considering the practical, physical and emotional perspective following discharge from hospital. Some of this could be linked to the clinician voice being strongly conveyed during this time due to the level of verbal information provided, and therefore, there is the possibility that the participants’ voice was not heard as equally. This concept has been explored in previous research studies (Val Linden et al., 2017; Hamilton et al., 2016) which recommend a shift in HNC consultations so that the patients’ voice is heard just as much as the clinicians, to ensure that patients’ values and preferences are incorporated into treatment decision‐making. A shift to using this approach in TL care could help future patients to fully consider their practical, physical and emotional needs in the longer term during this stressful and life‐changing time.

The majority of participants in this study were prepared for the practical and functional changes post‐operatively due to the practical information they were provided with pre‐operatively. However, some participants found it difficult to navigate their early adjustment to what ‘normal’ was for them post‐TL. This finding is supported by other qualitative research (Dawson et al., 2019) which describes how surgical HNC patients feel adequately prepared for the ‘physical’ changes following their surgery but found it challenging to navigate through the more emotional and psycho‐social adjustments to life after their surgery. This again links directly to the level and mode of pre‐operative information provision and decision‐making process; highlighting the need to re‐evaluate the structure and format of this intervention.

For participants who found it challenging to navigate the adjustment to the ‘new normal’, the motivation levels that they described during this time were low. Their ability to engage in activities such as stoma care competencies was often limited, so problem‐solving and navigating to a ‘new normal’ with this was difficult. Other research studies discuss issues with engagement (Neilson et al., 2013) and suggest that inpatient length of stay can increase as a result of low mood. This could be linked to those individuals’ ability to problem solve and adjust to their post‐treatment functional changes; particularly as the rates of psychological distress in people who have HNC are reported as being as high as 50% (Lydiatt et al., 2009). It is therefore possible that low levels of motivation reported by participants in this study could be similar to other research in HNC looking at mood. Early identification of people who experience low levels of motivation in the early post‐operative period could help to provide those individuals with increased support. This could help them to navigate more readily through their early post‐operative adjustment to the ‘new normal’.

This period of adjustment to the ‘new normal’ extended to participants first few weeks at home following hospital discharge. All participants highlighted the need for ongoing support at this stage to help with their transition home and coping with the life‐long adjustment to having a TL. This finding is supported by other qualitative work (Dunne et al., 2019; Nund et al., 2014) which highlighted the need for structured, individualized support from professionals, including emotional and psychosocial support in addition to support with physical changes. Participants in Nund's (2014) study talked about ongoing support coming from their own support networks of family and friends; whilst also raising the need for more specialist clinical support in the long‐term to help with their adjustment to life post‐treatment. This finding is similar to what participants in this study discussed as family support was key for them, but participants also highlighted the importance of ongoing specialist clinical support, with some participants discussing the disparity in community service provision. This potentially impacted on their coping and adjustment to life at home following TL and could be linked to a lack of experience, knowledge or time that community services have available. This is an area which should be explored in future research to deepen understanding of how services can be delivered to provide patients with optimum support at this time.

For participants in this study, the COVID‐19 era did not appear to have any influence to any of the four themes despite changes to the patient pathway. One small difference was described with regards to COVID‐19 in the ‘coping with adjustment to the new normal’ theme. Participants who had their surgery during COVID‐19 talked about the lack of face‐to‐face interaction with their family during their post‐operative hospital stay. Whilst participants commented on this being difficult from a lack of interaction perspective, the lack of visiting did not adversely impact on their adjustment to the ‘new normal’ when compared to participants who had their surgery before COVID‐19. Therefore, there is the possibility that changes to service provision due to COVID‐19 has impacted on clinician experiences and perspectives more than the patients; and this is something which could be explored in future studies investigating the impact of COVID‐19 on clinical services.

The aim of this study was to explore pre and during COVID‐19 inpatient experiences of having a TL. Some ERAS principles were incorporated into the pathway for participants who had their surgery during COVID‐19, for example, earlier contrast swallows post‐operatively to get back to eating and drinking sooner. However, information from participants in this study did not highlight any significant differences to experiences from pre‐operative preparation through to the early weeks at home following hospital discharge when compared to those who had surgery before COVID‐19. Although the current literature in ERAS advocates that early oral feeding positively influences patient recovery and outcomes (Seven et al., 2003; Prasad et al., 2006; Bannister & Ah‐See 2015; Aires et al., 2015); participants in this study focused more broadly, rather than focusing on specific time points, such as the timing of restarting oral intake. Participants talked more about how they were prepared for and adjusted to their functional changes post‐operatively, who supported them during this time and how this experience made them feel as a person. This feeling described by participants is supported by other research (Dawson et al., 2019; Santa Mina et al., 2021) in which physical, social, functional and psychological principles of care can maximize function; with people remembering how interactions made them feel rather than the details of the clinical interventions provided. This aligns more closely to what the participants in this study see as important in their TL pathway and could be considered for integration in future ERAS laryngectomy models of care.

Whilst this study generally reported positive experiences, there are limitations which should be considered. The participants in this study were recruited from a single centre. Experiences are therefore from one UK surgical centre and there may be subtle differences in service provision dependent on geographical location; for example, provision of community services available and individual centre care pathways. This was an exploratory study and results cannot be generalized widely and should be interpreted carefully. However, the demographics of the participants in this study are similar to the national demographic for HNC (NICE Improving Outcomes Guidance, 2014).

Findings from this preliminary study have highlighted potential avenues for further research. Participants in this study have talked about the need for specialist support beyond the hospital stay to support their longer term adjustment to the ‘new normal’. Future research could explore what these service models should look like in collaboration with patients, community service providers, that is, general practitioners and district nurses; and specialist hospital staff to shape future services for the benefit of the patient. There is also potential from this to re‐evaluate what ERAS services should look like for TL, extending to the pre‐treatment phase. Further work should be done to provide a patient‐centred ERAS model of care. There is potential that a model similar to the ‘multiphasic prehabilitation’ care package as described by Santa‐Mina (2020) would provide more than just information‐giving, and may help to prepare individuals for the longer term psycho‐social adjustment to life following TL. Future models of care should therefore consider both the format and content of information delivered to patients to maximize their outcomes. Tools to measure readability scores, for example, the Fog Index and co‐designing information packages with patients are potential options to improve future TL services.

This exploratory study has explored the acute experiences of patients undergoing a TL both before and during the COVID‐19 pandemic. There was no difference in experience reported by participants in this study who had their surgery either before or during COVID‐19 despite rapid service changes at their unit. Overall, people are adequately prepared for the functional changes they will face immediately following their surgery; however, there needs to be enhanced services provided to people having a TL both at the pre‐operative and early home discharge period.

Going forward, this study has shown how enhanced recovery could be instigated in clinical practice, and also demonstrated the need to shift enhanced recovery models of care away from the medical model alone. By making this shift there is potential to improve patient‐reported outcomes and achieve individualized care.

## CONFLICT OF INTEREST

No authors have no conflicts of interest to declare.

## ETHICS APPROVAL STATEMENT

This project received approval from the research and development (R&D) department at Sunderland Royal Hospital and Newcastle University.

FUNDING

The lead author received funding from CRN NENC for her MClinRes at Newcastle University for which this work was submitted as part of the final dissertation assessment.

## PATIENT CONSENT STATEMENT

All patients who participated in the study provided written consent.

## PERMISSION TO REPRODUCE MATERIAL FROM OTHER SOURCES

Yes.

## Supporting information

Supporting Information

## Data Availability

The data that support the findings of this study are available from the corresponding author upon reasonable request.
