# Supplementary material for: Patient experience of the acute post‐surgical period following total laryngectomy during the COVID‐19 era
Source: Int J Lang Commun Disord. 2022 Apr 11;57(4):737–48. doi: 10.1111/1460-6984.12709 (PMC9111097; doi:10.1111/1460-6984.12709)
Supplement: Supplementary file 1 — Supporting Information [file JLCD-57-737-s001.docx]

**Appendix 1 (patient information sheet and consent form; topic guide)**

**Information about the research**

**Study Title**

Patient experience of the acute post-surgical period following total laryngectomy during the COVID-19 era

**Invitation and brief summary:**

You have been invited to take part in this study as you have had surgery to remove your voice box within the last two years.

Joining this study is completely voluntary. We would like to give you some more information about the research.

A member of our team will go through this information sheet with you. They can answer any questions you may have. We expect this will take around 5-10 minutes. You can then decide whether or not you would like to take part.

Please feel free to ask questions at any time.

1. **What is the purpose and background to the research?**

This study is aiming to explore people`s experience of their time in hospital following surgery to remove their voice box both before and during COVID-19. We know that there have been major changes to the way we run our clinical services due to COVID-19 and would like to understand the impact that this has had on you. This will help us to know which part of our inpatient services are helpful, and allow us to make improvements to the service in the future.

1. **What would taking part involve?**

You will be invited to take part in an interview with us at XX Hospital. We aim to do this on a day that you are at the hospital for a clinic appointment. The interview will last between 30 – 45 minutes and will be audio-recorded.

1. **Why have I been invited to take part?**

You have been invited to take part because you had surgery to remove your voice-box within the last two years.

1. **What are the possible benefits of taking part?**

We do not expect that the study will directly benefit you; however, some people do find it helpful to talk about their experience in hospital. The information you provide in the interview about your experience will likely benefit the service we provide and our future patients.

1. **What are the possible disadvantages and risks of taking part?**

We do not expect there to be any disadvantages to taking part in the study. However, some people can find some topics difficult to talk about and may become emotional during the interview. If this was to happen, we will support you through this and can also offer further support from our Psychology service or signpost you to other avenues of support.

1. **How will my information be used?**

All information about participants will be handled in confidence. Completed interviews will be stored securely on an NHS computer in a password protected file that only XX can access. Names and addresses will not appear on any of our data. You will be given a unique number for identification and only XX will be able to link this number to your name and details.

The only other people allowed to know about who is in the study will be staff from the Research and Development Department of the hospital. They help to ensure that the research is being done properly. They will not disclose your identity to anyone else. We will keep the data from the study for ten years. Any identifiable data will be destroyed as soon as possible after the end of the study. All study records and participants’ rights to them will be protected in accordance with the UK data protection laws.

1. **What if something goes wrong?**

If you have any concerns about the study you can contact XX (contact details at the bottom of this Information Sheet) and she will do her best to answer your issue. If you remain unhappy and wish to complain formally, you can do this through the NHS Complaints Procedure. Details can be obtained from the hospital. If you would like to talk to someone not involved in the study, please contact the NHS Patient Advisory Liaison Service (PALS) Tel 0800 5876513. In the event that something goes wrong and you are harmed during the research due to someone’s negligence then you may have grounds for legal action for compensation but you may have to pay your legal costs. The normal NHS complaints mechanisms will still be available to you.

1. **What to expect during the consent process?**

After reading the study information, you will provide written consent to participate in the study. You will be given a copy of the consent form and information sheet to take home with you.

1. **What will happen if I don’t want to carry on with the study?**

You are free to withdraw from the study at any time. Coming out of the study will not change your future care in any way.

1. **What will happen to the results of the study?**

The results of the study will be written up for a Masters research Dissertation submitted to XX. They may also be shared at national and international research conferences and submitted to an academic journal for publication.

A summary of the study will be available to research participants. You will not be identified by name in any reports or publications.

1. **Who is organising and funding the study?**

The study is being led by XX. XX`s research time is being funded by the XX. The XX have no role in the study design, data collection/analysis/interpretation or report writing.

1. **Who has reviewed the study?**

This study has been reviewed by the Research Design Service at XX

***Further information and contact details***

For further information regarding the study, please contact:

XXX

Thank you for taking the time to read this information sheet.

**CONSENT FORM**

Centre: XX

Participant Identification Number for this trial:

Title of Project: Patient experience of the acute post-surgical period following total laryngectomy during the COVID-19 era

Name of Researcher: XX

Please initial box

1. I confirm that I have read the information sheet dated for the
   above study. I have had the opportunity to consider the information, ask questions and have had these answered satisfactorily.
2. I understand that my participation is voluntary and that I am free to withdraw at any time
   without giving any reason, without my medical care or legal rights being affected.
3. I understand that relevant sections of my medical notes and data collected during
   the study may be looked at by individuals from XX, where it is relevant to my taking part in this research. I give permission for these individuals to have access to my records.
4. I understand that the information collected about me may be used to support
   other research in the future, and may be shared anonymously with other researchers.
5. I agree to take part in the above study.

Name of Participant Date Signature

Name of Person Date Signature

taking consent

**Topic guide**

Introduction

1. Introduce self and thank for agreeing to participate

2. Participation is optional and you can stop the interview or decline to answer specific questions if you wish. It should last no longer than an hour.

Confidentiality and consent

1. The findings may be written up in the service evaluation but you will be remain anonymous

2. I would prefer to record the interview to help capture everything you say and also to evaluate the questions that I use. I may repeat some of your answers to make sure I have understood you. Everything you say will remain on a device that can only be accessed by password and transcribed on a password protected NHS computer. Is this okay with you?

3. Clarify that they understand the purpose and confidentiality of the service evaluation and that they are happy to partake. Consent form signed.

4. Any questions.

These questions are about general information, giving me an idea of the background of your laryngectomy.

This is about your experience when you were in hospital having your laryngectomy – what jumps out for you? What were the low points? Was there anything that particularly went well?

Probe further dependent on participant response

Before you came in to hospital for your operation – what was that like for you? (probe for level of preparation e.g. pre-treatment information, including setting; materials to support info; meeting another patient)

Why did you feel like that? Is this the information that you wanted to know? (probe dependent on response)

How did you think you would cope with the surgery and life afterwards? Why?

Communication

Tell us about how you communicated after your surgery … what was that like for you and others?

What helped / what hindered

Why?...

Socialising with others

What was it like taking to other people on the ward?

What was it like talking to staff?

How did you communicate with your family? (may need to probe around lack of visitors)

(If you can, get them to describe an experience of trying this out – anything that helped or hindered?)

Why?....

Eating and drinking

Tell us about your experiences of eating and drinking after your operation?

(probe whatever they come up with .. if not very much, ask about having a tube in .. can they recall the first attempt at eating; how long after their surgery was this – try to get some detail, how did it feel?)

Why?....

Stoma care

Tell us about your stoma – what was your experience of it in hospital? How did you get to learn to look after it – what helped? What hindered? Probe whatever they come up with – try to get some info on timings

Why?....

Mobility / dressing

Can you tell us a bit more about getting back to doing everyday things like dressing and washing, moving around? How was this for you – any problems? Anything that went better than expected?

Why did you/did you not do that? Why did you feel like that?

Discharge

Can you tell us a bit more about your discharge from hospital – anything that particularly bothered you about going home? How did this actually go? How many days were you in hospital?

What was it like for you in the first few days/weeks at home?

Why???

Experience

1. How would you describe your overall experience? Specifically of having a laryngectomy during covid-19 pandemic (if during this timeframe)?

2. Was there anything that could be done differently? Why?

3. How did it make you feel? Why?

4. What went well for you? Why?

5. How did it make you feel? Why?

Summarising and reflecting

Just thinking about your hospital stay after you lary – is there anything else you want to talk about?

Is there anything else you would like to add?
